# Effect of Fentanyl Addition to Local Anaesthetic in Peribulbar Block

**Published:** 2009-02

**Authors:** Mostafa Abdel Hamid Abo El Enin, Ismail Ewis Amin, Ahmed Sayed Abd El Aziz, Mostafa Mohamed Mahdy, Mohamed Abdel Hamid Abo El Enin, Mostafa Mahmoud Mostafa

**Affiliations:** 1 Lecturer in Anaesthesia, Department of Anesthesia and Intensive Care and Ophthalmology, Faculty of Medicine, Al-Azhar University, Egypt; 2 Lecturer in Anaesthesia, Department of Anesthesia and Intensive Care and Ophthalmology, Faculty of Medicine, Al-Azhar University, Egypt; 3 Lecturer in Anaesthesia, Department of Anesthesia and Intensive Care and Ophthalmology, Faculty of Medicine, Al-Azhar University, Egypt; 4 Prof. in Anaesthesia, Department of Anesthesia and Intensive Care and Ophthalmology, Faculty of Medicine, Al-Azhar University, Egypt; 5 Lecturer in Ophthalmology, Department of Anesthesia and Intensive Care and Ophthalmology, Faculty of Medicine, Al-Azhar University, Egypt; 6 Lecturer in Ophthalmology, Department of Anesthesia and Intensive Care and Ophthalmology, Faculty of Medicine, Al-Azhar University, Egypt

**Keywords:** Fentanyl, Local anaesthetic, Peribulbar block

## Abstract

**Summary:**

Forty patients ASA I, II undergoing vitrectomy due to vitreous hemorrhage not associated with retinal detachment were divided into two groups randomly, each of them with 20 patients. In Control group patients received local anaesthetic only, while Fentanyl group receive 20 mcg fentanyl added to local anaesthetic, the onset and duration of lid and globe akinesia were assessed at 1,3,5 and 10 min. Postoperative VAS was recorded each hour up to 6^th^ hour.

The results show statistically significant difference between the two groups in the onset of lid akinesia. Fentanyl group had faster onset of lid akinesia and had significantly longer duration of akinesia (196.5 ± 14.24 min). There is statistically significant difference between the two groups in the onset of globe akinesia at 3, 5 min. Fentanyl group had faster onset than Control group and had longer duration of globe akinesia (294 ± 17.89 min).

Fentanyl group had prolonged duration of analgesia 3.25±0.67 hr as compared to 1.85±0.67 in Control group, *P*=0.00 postoperatively. There were statistically significant differences between the two groups as regard the mean VAS in 1,2,3,4 hours, Fentanyl group had lower median pain score than Control group. Addition of fentanyl to local anaesthetic mixtures fasters the onset and prolong the duration of akinesia and improve quality of postoperative pain in peribulbar block.

## Introduction

Fentanyl (N-phenyl-N-(1-Phenethyl-4-piperi-dinyl) propanamide) is anopioid analgesic with potency eighty times that of morphine.

Fentanylis extensively used for anaesthesia and analgesia in the operating room and intensive care unit. It is frequently given intrathecally as a part of spinal anaesthesia or epidurally as apart of epidural anaesthesia and analgesia, it is also used as a sedative.^1^ Addition of small amount of local anaestheics augments the effect of intrathecal opioids by increasing the duration of the block and speeding the onset of analgesia.^2^

Fentanyl is added commonly to local anaesthetic administrated in the extradural space to improve analgesia in the postoperative period.^3^ The addition of fentanyl produced only slight change in the quality and duration of analgesia after administration of 2% lidocaine with epinephrine for a short surgical procedure^4^ or after administration of 0.125% bupivacaine^5^, other studies^6^ in adults report improved and/or prolonged analgesia following the addition of fentanyl to lumbar extradural bupivacaine for lower abdominal procedures, caesarean section and pain relief in labour.^7^

This study is designed to examine the effect of adding fentanyl to local anaesthetics in peribulbar block on the onset and duration of lid and globe akinesia and postoperative analgesia.

## Methods

After institutional approval and informed consent, 40 adult patients of both sexes with ASA grade 1-2, scheduled for vitrectomy due to vitreous hemorrhage were divided into two groups each of them 20 patients randomly by using a table of random numbers and sealed closed envelopes in a randomized fashion.

Complete ophthalmological examination was done by the ophthalmologist as well as ophthalmic ultrasound and biometry was done for all cases to exclude complicated vitreous hemorrhage, diagnosis of any associated disorders and diagnosis of posterior staphyloma if it was present, also the axial length was measured.

## Exclusion criteria

1. Patients with impaired orbital/periorbital sensation.
2. Patients having history of abnormal bleeding or allergy to local anaesthetics.
3. Patients with complicated vitreous hemorrhage as retinal detachment, extensive epiretinal membranes, drooped nucleus or IOL, as such surgery takes a long time or when the surgeon expected prolonged surgery (= 2 hrs).
4. Patients with posterior staphyloma.
5. Patients with axial length more than 28 mm.

Control group: Volume of 6-10 ml (5 ml mepivacaine 3% + 1 ml hyaluronidase (150 mcg) + 3 ml bupivacaine 0.5% + 1 ml saline) was used.

Fentanyl group: Volume of 6-10 ml (5 ml mepivacaine 3% + 1 ml hyaluronidase (150 mcg) + 3 ml bupivacaine 0.5% + 1 ml saline containing 20 mcg fentanyl) was used.

After securing intravenous access, 1 mg midazolam was given intravenous with 25 mcg fentanyl and topical anaesthesia in the form of tetracaine eye drops 0.5% applied to both groups. 2 ml lidocaine 2% diluted with 13 ml saline to form mixture for painless local injection^8^. 1 ml from these mixture was given transconjunctival in the medial canthus in the tunnel (between the caruncle and the medial canthal angle) with insulin needle1cm length 27G, then 3-5 ml of local anaesthetic mixtures according to each group was injected with needle 27G and 3 cm length with angle 45° between the caruncle and medial canthal angle till the tip of the needle touch the ethmoid bone then the direction of the needle changed to 90° with the hub of the needle at thelevel of the iris. Other 3-5 ml from local anaesthetic mixture was injected in the extreme inferotemporal border of the orbit with the same needle 27G and 3cm length directed downward and medially below the globe. Light orbital compression for 1 minute then evaluation after 1 minute, 3 min, 5 min, and 10 minute. The appearance of proptosis and chemosis was observed immediately after the block. The onset and duration of lid and globe akinesia were assessed every 1 minute until maximum blockade and then every 15 minutes after surgery until complete recovery of the block.

## Evaluation of the block

Motor block evaluation include slid akinesia (lid closure by orbicularis and lid opening by the levator) and globe akinesia using 3 point scale for every muscle was done using the score system that shown in Table 1.

**Table 1:** Scoring system:^9^

Akinesia of extraocularmuscles including the levatormuscle.
**0** = 0-1 mm movement in 1 or 2 main directions. or 0 to 4 mm movement in levator muscle.
or 0 to 4 mmmovement in levatormuscle.
**1** =1 mm movement in more than 2 main directions or 2 mm movement in any main direction or more than 4 mm movement in levator muscle.
**2** = > than 2 mm movement in any main direction or 2 mm movement in 2 or more main direction
Akinesia of orbicularismuscle.
**0**= Complete akinesia.
**1**= Partial movement in either or both eyelid margins.
**2**= Normal movement in either or both eyelid margins.

For assessment of lid akinesia the patients were asked to open their eye lids and then squeeze them together maximally. Orbicularis occuli muscle was assessed separately by using the score in Table 1. Also levator palpebrae muscle for opening eye lid was assessed by the score in Table 1. Globe akinesia was assessed at 1 minute, then 3 minute, 5 minute, 10 minute and 15 minute. These were scored using the movements of the extra ocular muscles in all 4 main directions on a scale of 0 to 2 as shown in Table 1. The block was considered satisfactory when loss of at least two movement of the 4 cardinal direction.^10^

Arterial blood pressure, heart rate and oxygen saturation (SpO2) were checked every 15 minutes during the entire procedure and every 30 minutes during the first two postoperative hours. Hypotension and bradycardia were defined as a 20% decrease in blood pressure and heart rate in relation to preblock value.

Postoperative analgesia was assessed by using Visual Analogue Score (VAS) every hour upto 6 hours postoperatively as 0 (no pain) to 10 (maximum pain imaginable). If the VAS was >5, injection of diclofenac 75 mg intramuscular was given. Enquiry was made about any adverse effect such as nausea, vomiting, dryness of mouth, dizziness, diplopia and blindness.

## Statistical analysis of data

Statistics were done by computerusing Epi-info. Software version 6.04. A word processing, database and statistics program (WHO, 2001). The tests used were: X(mean), SD(standard deviation): to measure the central tendency of data and the distribution of data around their mean value. Student's t test: for testing statistical significant difference between mean values of two samples. X^2^ test (Chisquare test) to test for statistical significant relation between different variable or grades in qualitative data. ANOVA or F test: to test for significant difference between more than two samples mean values. Mann Whitney test: non parametric test for comparing two groups of data not normally distributed or for small sample size. Fisher exact test: for comparing two independent proportions when the expected observation in any cell of the table is below 5. Significant result is considered if *P* < 0.05. Highly significant resultis considered if *P* < 0.01.

## Results

There was no statistical significant difference between the two groups in the general characteristics including age, sex, weight, volume or in duration of surgery as shown in Table 2.

**Table 2:** General characteristics of the studied groups

Variable	Control	Fentanyl	*P*value
No. of cases	20	20	
Age(year)	55.55±6.29	60.15±6.25	0.1082
Male n(%)	14(70)	16(80)	0.3966
Female n(%)	6(30)	4(20)	
Weight(kg)	75.95±9.46	80.9±8.46	0.1871
Volume injected(ml)	7.7±0.98	8±0.97	0.6883
Duration of surgery(min)	93.25±10.56	89.55±6.97	0.2016

Data aremean ± SD. Statistically significant *P value < 0.05 highly significant ** P value<0.001

- The result of eye lid closure akinesia was the same as result of eye lid opening akinesia and showed statistically significant difference between the two groups in the onset of lid akinesia at 5 min (no patients in Fentanyl group remained to 5 min while in Control group 3 patients remained and got complete akinesia at 5 min). So fentanyl group had a statistically significantly faster onset of lid akinesia than controlgroup.
- There was statistically significant difference between the two groups in the duration of lid akinesia fentanyl group have prolonged duration of akinesia but faster onset as shown in Table 3.
  **Table 3:** Comparison between Fentanyl group and the Control group in eye lid akinesia having grade 0 akinesia(complete akinesia)

Lid akinesia	Control group(20)		Fentanyl group(20)		*P*
	No.	%	No.	%	
**Onset**					
1min.	1	5	4	20	0.1514
3min.	15	75	16	80	0.7049
5min.	3	15	0	0*	0.0117
10min.	1	5	0	0	0.3111
Duration of lid akinesia(min)	122.25 ±10.82		196.5±14.24**		0.0000

Statisticallysignificant

* *P* < 0.05 highlysignificant

** *P*<0.001
- There was statistically significant difference between the two groups in the onset of globe akinesia at 3,5 min, 3 patients (15%) in Control group got a complete akinesia at 3 min and 14 patients (70%) at 5 min; while in Fentanyl group 13 patients (65%) and 6 patients (30%) respectively.
- There was statistically significant differences between the two groups in the duration of globe akinesia Fentanyl group have long duration in akinesia but faster onset (Table 4)
  **Table 4:** Comparison between Fentanyl group and the Control group in globe akinesia (EOM) having grade 0 akinesia (complete akinesia)

Globe akinesia	Control group(20)		Fentanyl group(20)		*P* value
	No.	%	No.	%	
**Onset**					
1min	0	0	0	0	0
3min	3	15	13	65**	0.0012
5min	14	70	6	30*	0.0114
10min	3	15	1	5	0.2918
15min	0	0	0	0	0
**Duration of globe akinesia(min)**	185±11.92		294 ±17.89**		0.0000

Statisticallysignificant

* *P* value< 0.05 highly significant

** *P* value <0.001
- There was highly significant difference between the two groups in first time to require analgesia. In the first hour 30% of patients (n=6) required analgesia and 55% (n=11) in the second hour but no patients required analgesia in Fentanyl group but 75% (n=15) patients in Fentanyl group required analgesia after 3 hours (Table 5).
  **Table 5:** Comparison between Fentanyl group and the Control group in time for first analgesic request.

1^st^ analgesic requirement	Control group(20)		Fentanyl group(20)		*P* value
	No.	%	No.	%	
1 hr	6	30	0	0**	0.0078
2 hr	11	55	0	0**	0.0000
3 hr	3	15	15	75**	0.0001
4 hr	0	0	5	25	0.3111
Mean ± SD(hr)	1.85±0.67		3.25 ±0.44**		0.0000

Statistically significant *P value< 0.05 highly significant

** *P* value <0.001
- There was statistically significant differences between the two groups as regard the median VAS at 1, 2, 3, 4, 5, 6 hours Fentanyl group had lower median pain score than Control group. Postoperative analgesia given in both groups when VAS>5 (Table 6).
  **Table 6:** Postoperative VAS for the studied groups

Postop.VAS	Control(n=20)	Fentanyl(n=20)	*P*
1^st^ hr	2(2–3)	2(1–2)*	0.000
2^nd^ hr	5(4–6)	4(3–4)*	0.000
3^rd^ hr	6(6–7)	6(5–7)*	0.000
4^th^ hr	4(3–6)	3(2–5)*	0.000
5^th^ hr	4(2–5)	4(2–5)	0.9795
6^th^ hr	4(2–5)	4(2–5)	0.9795

* = statistically significant values are median (range)
- We did not observe any side effect during the study related to peribulbar block, there were no statistically significant differences in peripheral oxygen saturation, heart rate and non invasive blood pressure between the two groups.

## Discussion

Opiates are widely known to have an antinociceptive effect at the central and/or spinal cord level^11^. However, evidence has begun to accumulate that opioid antinociception can be initiated by activation of peripheral opioid receptors^12^. The presence of peripheral opioid receptors is shown in immune cells and primary afferent neurons in animals.^13^ If opioid administration improves regional anaesthesia without centrally mediated side effects, it would be useful in clinical practice.

Study has demonstrated the presence of peripheral opioid receptors that mediate analgesia by endogenous as well as exogenous opioid agonists.^14^ It is speculated that the peripheral administration of opioids provides tronger and longer lasting analgesia with a lower dose of opioid without central side effects such as respiratory depression, nausea, vomiting and pruritus.^15^ A number of trials have examined the peripheral analgesic effect of opioids in a large variety of surgical settings particularly arthroscopy and conduction nerve blocks.^16^,^17^

The addition of opioids in brachial plexus block is reported to improve success rate and postoperative analgesia.^18^ We postulate the possible mechanisms of action for the improved analgesia produced by the peripheral application of fentanyl. First, fentanyl could act directly on the peripheral opioid receptor. Primary afferent tissues (dorsal roots) have been found to contain opioid binding sites^13^. Because the presence of bidirectional axonal transport of opioid binding protein has been shown^19^ fentanyl may penetrate the nerve membrane and act at the dorsal horn. This could also account for the prolonged analgesia. However, fentanyl is reported to have a local anaesthetic action.^20^ Gormley et al^18^ suggested that alfentanil also prolonged postoperative analgesia by local anaesthetic action.

Second, fentanyl may potentiate local anaesthetic action via central opioid receptor-mediated analgesia by peripheral uptake of fentanyl to systemic circulation.^21^

Whether fentanyl diffuses from the peribulbar space to the subarachnoid space around the optic nerve in the reterobulbar space or not to clarify this issue, the spinal fluid fentanyl concentrations should be measured.

A synergistic interaction between local anaesthetics and opioids with epidural administration has been reported.^22^ It appears that local anaesthetics and opioids exert their action independently via different mechanisms. Local anaesthetics block propagation and generation of neural action potentials by a selective effect on sodium channels, whereas opioids act on the opioid receptors creating an increase in a potassium conductance. This action results in hyperpolarization of then erve cell membrane and a decrease in excitability^23^. Although sodium channel block is proposed to be the primary mode of action, local anaesthetics also have an effect on synaptic transmission^24^. Li et al^24^ showed that lidocaine inhibited both substances P binding and substance P-evoked increase in intracellular calcium. In contrast, in addition to the considered primary mode of action, opioids were found to directly suppress the action potential in nerve fibers^25^. Frazier et al^26^ showed that morphine depressed both sodium and potassium currents associated with the action potential in squid giant axons. Therefore, the combination of local anaesthetics and opioids may effectively inhibit multiple areas of neuronal excitability.

The addition of hyaluronidase to local anaesthetic mixtures decreases the onset time of peribulbar block and quality of akinesia in most reported studies.^27^

The present study compares the effect of addition of fentanyl to local anaesthetic mixtures in peribulbar block on the onset and duration of complete akinesia. The results of the present study showed that addition of fentanyl to local anaesthetic mixtures in peribulbar block fasten the onset of block (80% of patients get complete lid akinesia at 3 min and no patients remained to 5 min while in control group 15% get complete lid akinesia at 5 min and 5% at 10 min.). Also Fentanyl group had a short onset in globe akinesia 65% at 3 min, 30% at 5 min and only 5% at 10 min but in Control group 15% at 3 min, 70% at 5 min and 15% at 10 min. As regard duration of lid and globe akinesia Fentanyl group had longer duration than control group. The results of the present study are in accordance with study done by Toshiharu et al^28^ who studied the effect of addition of fentanyl to mepivacaine in epidural block and found that addition of fentanyl to mepivacaine accelerate the onset of analgesia and enhances the analgesic effect of epidural block. Deniz et al^29^ found that addition of fentanyl to bupivacaine in brachial plexus axillary approach prolong anaesthesia and analgesia, prolong duration of sensory and motor block and prolong the duration of postoperative analgesia. In the present study the first time to require analgesia is prolonged in Fentanyl group, in which 75% of patients required rescue analgesia 3 hour postoperative while in Control group 30% required it in first hour and 55% in second hour and 15% in the third hour. These results are similar to the results of constant O et al^30^ who studied the effect of addition fentanyl to local anaesthetic mixture in caudal block in children undergoing bilateral vesicoureteral reflex and they found that addition of fentanyl (1 mcg.kg^−1^) to bupivacaine 0.25% and lidocaine 1% prolong duration of surgical analgesia after single injection from start of injection to first requirement of analgesia from 174 min in Control group to 253 min in Fentanyl group. In the present study fentanyl group had lower pain score postoperatively than in Control group. In accordance with the study done by Vita et al^31^ who found that intraarticular injection of fentanyl improve postoperative pain and no difference between intraarticular morphine and fentanyl in postoperative pain relief.

Also Vijay et al^32^ found that wound infiltration with fentanyl has lower VAS postoperatively and combination of lidocaine with fentanyl for wound infiltration in cholecystectomy patients was associated with better postoperative analgesia, reduced analgesic consumption and better lung function. Saryazdi et al^33^ found that injection of fentanyl intraarticularly has better postoperative pain less pain score and short time to walk were achieved by fentanyl orpethedine in comparison with dexamethasone when injected intraarticular.

The present study concluded that addition of fentanyl to local anaesthetic mixtures in peribulbar block fasters the onset and prolong duration of lid and globe akinesia, and improves quality of analgesia.
